# Apparent total tract and ileal amino acids digestibility values of vegetal protein meals with dietary protease to broiler diet

**DOI:** 10.1093/tas/txaa219

**Published:** 2020-11-27

**Authors:** Antonio G Bertechini, Felipe Santos Dalolio, Julio C C Carvalho, Andressa C Carvalho, Jose O B Sorbara

**Affiliations:** 1 Animal Science Department, Federal University of Lavras, Minas Gerais, Brazil; 2 DSM Nutritional Products, São Paulo, Brazil

**Keywords:** corn, dietary enzyme supplementation, ileal amino acids digestibility, full-fat soybean meal, soybean meal

## Abstract

This experiment was carried out to study the effect of dietary exogenous monocomponent protease on the coefficient apparent total tract (ATTD) and apparent ileal (AID) digestibility of amino acids of corn, soybean meal (SBM), and full fat soybean meal (FFSM) in broilers. A total of 400 males Cobb-500 (14 d of age) were equally allocated in 80 metabolic cages (50 cm × 50 cm × 45 cm) in a completely randomized design and a semi-controlled environment. Eight treatments (basal diet with or without a protease and three ingredients replacing the basal diets in 40% to corn and 30% to SBM and FFSM, with and without protease), with 10 replicates each were evaluated. The protease was added at 200 mg/kg resulting in 15,000 unit of PROT/kg. The total collection of excreta was held during 3 d, after 5 d for adaptation of broilers at the diets. The ileal content was collected on d 21, after slaughter of birds. The enzyme increased (*P* < 0.05) the ATTD and AID of most amino acids contained in SBM and FFSM. The digestibility of cysteine, glycine, proline, and threonine had higher (*P* < 0.05) ATTD and AID in all tested ingredients with the use of protease. On average, the dietary protease increased in 5.19% and 3.86% the total and ileal digestibility of amino acids, respectively. It was concluded that the dietary protease exerts major effects on toasted full-fat soybean, followed by soybean meal and corn to broilers.

## INTRODUCTION

Most poultry diets are formulated with vegetable ingredients such as corn, soybean meal (SBM), and toasted full-fat soybean meal (FFSM), being these ingredients considered primary in America. There is a large knowledge of the contents of metabolizable energy and amino acids digestibility of these feedstuffs. In general, they have digestibility of amino acids ranging from 80% to 90% (NRC, 1994; Rostagno et al., 2017). Although these ingredients are considered high in protein digestibility, there is still a portion of nutrients, particularly amino acids, that are not used during bird´s metabolism and can contribute for excretion of nitrogen to the environment (Stefanello et al., 2016; Cowieson et al., 2018).

Differences on ileal digestibility between feedstuffs are common in the literature. Coefficients of apparent ileal digestible (AID) for lysine contained in SBM, for example, can vary of 0.810 to 0.901 in diets with no enzyme addition. For FFSM the variations are greater, from 0.798 (Clarke and Wiseman, 2005) to 0.856 (Rostagno et al., 2017), due to the type of processing and fat content of this feedstuff. These difference between ingredients of diets can influence the efficiency of meat production of broilers.

The industrial protease production caused expectations for its use since amino acids represent high costs in the diets for animals. In broilers, due to intensive breeding, the dietary nutritional needs are higher, mainly of amino acids. Thus, the use of exogenous enzymes such as protease may contribute to the increased efficiency of use of dietary protein.

Studies with proteases in diets with several ingredients for broilers have shown increase in digestibility of methionine, cysteine, histidine, valine, isoleucine, leucine, serine, glycine, and proline (Tejedor et al., 2001; Kocher et al., 2003; Bertechini et al., 2009; Carvalho et al., 2009; Angel et al., 2011; Freitas et al., 2011; Kamel et al., 2015). In order to contribute to the upgrading of chemical composition tables and digestibility of feedstuffs for broilers, the objective of this study was to evaluate the effect of dietary monocomponent protease on coefficient of apparent total tract digestibility (ATTD) and AID of amino acids of corn, SBM and FFSM in broilers diets.

## MATERIAL AND METHODS

All experimental procedures in this study were approved by the Committee for Use of Animals in Research of the Federal University of Lavras, Minas Gerais, Brazil.

### Birds and Housing

A total of 400 male Cobb-500 chicks of 1 d of age, obtained from commercial hatchery, properly vaccinated against Marek’s were used. The birds were raised in the litter (wood shavings) from 0 to 13 d of age, feed with corn–soybean meal diet formulated according to the recommendations of Rostagno et al. (2017). At 14 d of age, the birds (488 ± 10 g of live weight) were randomly placed into 80 metabolism cages (50 cm × 50 cm × 45 cm) with controlled environment (average of 24.8 ± 1.3 °C of temperature, 65.4 ± 2.1% of relative humidity and 24L:0D light program). The cages were equipped with individual feeder, nipple drinkers and metal tray for excreta collection. Feed and water were provided for ad libitum during all experimental period (14–21 d).

### Experimental Procedures

Eight treatments (basal diet with or without a protease and three ingredients replacing the basal diets with and without protease), with 10 replicates each were evaluated. The replacement of basal diet for the tested ingredients were 40% for corn and 30% for SBM and FFSM (Matterson et al., 1965). The protease used was Ronozyme ProAct 75,000 PROT U/g (EC. 3.4.21, DSM Nutritional Products, Basel, Switzerland), an alkaline serine hydrolase derived from *Nocardiopsis prasina* and produced by *Bacillus licheniformis*. The protease was added to diets a expense of the inert (kaolin) at 200 mg/kg, resulting in 15,000 unit of protein U/kg. The analyzed basal diet and the ingredients were in Table 1.

**Table 1.** Analyzed composition and mean particle size (MPS) of basal diet and ingredients used in the study (as-fed basis)^*a*^

Item (g kg^‒1^)	Basal diet	Corn	SBM	FFSM
Dry matter, g kg^‒1^	899.5	880.1	892.6	894.8
Crude protein, g kg^‒1^	210.5	78.0	458.7	325.8
Crude fat, g kg^‒1^	55.3	37.7	16.5	208.3
Crude fiber	35.3	16.8	57.5	56.0
Ash, g kg^‒1^	28.2	13.2	49.8	43.8
Urease activity		–	0.05	0.07
Gross energy, MJ kg^‒1^	12.6	16.2	17.1	21.1
Amino acids, g kg^‒1^				
Alanine	9.5	5.8	18.7	15.9
Aspartic acid	17.2	3.1	30.9	26.8
Arginine	12.5	3.5	31.0	27.8
Cysteine	3.7	1.8	5.5	5.7
Glycine	7.9	3.8	18.7	15.2
Glutamic acid	33.9	5.4	53.9	36.4
Histidine	4.8	1.9	10.8	9.8
Isoleucine	8.3	2.9	20.7	16.6
Leucine	16.2	8.8	33.7	27.5
Lysine	12.3	2.4	28.1	23.2
Methionine	4.7	1.7	6.4	4.6
Phenylalanine	9.3	3.5	23.2	18.1
Proline	11.6	7.8	22.7	19.0
Serine	8.9	2.9	24.1	18.5
Threonine	7.6	3.0	18.6	14.5
Tyrosine	7.4	2.2	16.7	12.5
Valine	10.0	3.8	22.6	18.5
Total AA	185.8	73.5	418.1	288.2
MPS, µm ± sd	755±7	765±5	785±8	727±7

^*a*^Amino acid analyzed by HPLC method.

The total collection of excreta was held during 3 d (19, 20, and 21 d of age of broilers), after 5 d for adaptation of broilers at the diets. The collections were made twice a day, in order to avoid possible fermentation of excreta. The beginning and end of the collection were scored using the ferric oxide (1%) in the diet. Marked excreta were the reference for the beginning and ending of period of collection. The collected excreta were placed in pre-labeled plastic bags and stored in a freezer at ‒4 °C until the end of the experimental period. Then, the samples were thawed, weighed, homogenized, and aliquots of 300 g were predried in oven with forced ventilation at 55 °C during 72 h. Subsequently, they were weighed and milled in an analytical mill “Willey” (Tecnal, Croton TE-625, Piracicaba, São Paulo, Brazil), together with the diets and tested ingredients for laboratorial analysis. The gross energy (GE) was determined using a calorimeter (model 1261, Parr Instrument Company, Moline, IL, USA) and expressed in MJ GE/kg natural matter. Analyses of dry matter and crude protein (CP) were made according to the methodology described in AOAC (2000) and analysis of AA made in HPLC (SHIMADZU, Model LC-10AT, Shimadzu Corp., Kyoto, Japan) methodology (White et al., 1986; Hagen et al, 1989).

For determination of AID of amino acid, with 24 h prior to collection of the ileum of broilers, diets were mixed with 1% Celite marker (Scoot and Boldaji, 1997). This indigestible marker was provided to the animals until the time of slaughter. At 21 d of age, four birds of each cage were slaughtered by cervical dislocation to obtain the ileal content. Immediately after slaughter, the ileum was exposed by abdominal incision and a segment of 15 cm, starting at 1.0 cm from the ileo-cecal junction, was sectioned. With a light hand pressure, the content of this segment was collected in plastic containers properly identified, sealed, and then stored in a freezer at ‒4 °C. Posteriorly, the ileal contents were lyophilized under vacuum and at a temperature of ‒40 °C for 72 h. After this, samples were macerated and forwarded for analysis of AA and acid-insoluble ash (Scoot and Boldaji, 1997) to determine the values ​​of AID of amino acid.

### Digestibility Procedures of Amino Acids

All digestibility coefficients of amino acids were determined on dry matter basis. ATTD and AID were calculated as follows:

$$\begin{array}{ll} ATTD_{AA}= & \;\;ATTD_{AAbasal\;diet}\\ & +\frac{(ATTD_{AAtest\;diet}-ATTD_{AAbasal\;diet})}{Level\;of\;inclusion,\;\%} \end{array}$$

$$\begin{array}{l} AID_{AA}=\;AID_{AAbasal\;diet}+\frac{(AID_{AAtest\;diet}\;-\;AID_{AAbasal\;diet})}{Level\;of\;inclusion,\;\%} \end{array}$$

$$\begin{array}{l} AID\;=\frac{\left[\left(AA/Celite\right)d-\left(AA/Celite\right)i\right]}{\left(AA/Celite\right)d} \end{array}$$

where ATTD is the apparent total tract digestibility of individual AA (%), AID is the apparent ileal digestibility of individual AA (%), (AA/Celite)_d_) is the dietary ratio of amino acid to Celite and (AA/Celite)_i_ is the ratio of amino acid to Celite in ileal digesta.

The differentials of the enzyme effects on each ingredient expressed as a g kg^‒1^ were calculated.

### Statistical Analysis

To compare the effect of dietary enzyme supplementation for each tested ingredient, data were analyzed by ANOVA using the General Linear Models procedure SAS (2004). Differences were considered significant when *P* < 0.05 and means were compared by the Least Significant Difference test.

## RESULTS

The dietary protease increased (*P* < 0.05) the coefficient of ATTD and AID of amino acids in all tested ingredients, however, in a different way. In corn, there were increases in ATTD, more specifically to cysteine, glycine, isoleucine, proline, serine, threonine, and valine (Table 2). In general, increases in ATTD range from 4.05% to 11.7% between the different amino acids. For coefficient of AID, the dietary protease increased (*P* < 0.05) increases range from 2.32% to 8.28%. The greatest increases in the ATTD were observed for glycine (11.7%) and the threonine (9.9%), while for AID, the greatest increases occurred with glycine (8.3%) and serine (7.4%).

**Table 2.** Coefficients of ATTD and AID of amino acids of corn with or without dietary protease

Amino acids	ATTD^†^			AID^†^		
Protease, ppm	0	200	SEM	0	200	SEM^*a*^
Alanine	0.793	0.828	0.0137	0.823	0.835	0.0071
Arginine	0.815	0.836	0.0122	0.837	0.844	0.0131
Aspartic acid	0.845	0.846	0.0065	0.902	0.911	0.0077
Cysteine	0.750^b^	0.792^a^	0.0053	0.836^b^	0.855^a^	0.0088
Glutamic acid	0.907	0.918	0.0042	0.896	0.901	0.0056
Glycine	0.659^b^	0.737^a^	0.0110	0.687^b^	0.744^a^	0.0155
Histidine	0.855	0.860	0.0031	0.884	0.907	0.0076
Isoleucine	0.822^b^	0.852^a^	0.0033	0.857^b^	0.877^a^	0.0079
Leucine	0.883	0.895	0.0029	0.874	0.887	0.0067
Lysine	0.878	0.880	0.0022	0.872	0.882	0.0086
Methionine	0.873	0.875	0.0050	0.872	0.884	0.0036
Phenylalanine	0.871	0.881	0.0026	0.900	0.910	0.0056
Proline	0.825^b^	0.860^a^	0.0053	0.843^b^	0.881^a^	0.0076
Serine	0.795^b^	0.833^a^	0.0049	0.832^b^	0.894^a^	0.0088
Threonine	0.722^b^	0.793^a^	0.0085	0.813^b^	0.871^a^	0.0105
Tyrosine	0.869	0.878	0.0033	0.919	0.934	0.0078
Valine	0.814^b^	0.847^a^	0.0071	0.799^b^	0.824^a^	0.0096
Total AA	0.838^b^	0.860^a^	0.0031	0.851^b^	0.874^a^	0.0045
Total × ileal^‡^	0.839^B^			0.865^A^		

^†^Different lower case letters within a row indicate significantly different means by least significant difference test (*P* < 0.05).

^‡^Different capital letters in the line indicate significantly different means by *F* test (*P* < 0.05). The data is from 10 replicates.

^*a*^SEM: standard error of the mean.

For SBM, protease supplementation increased (*P* < 0.05) the ATTD and AID of total amino acids, more specifically of arginine, cysteine, glycine, histidine, lysine, methionine, proline, threonine, tyrosine, and valine (Table 3). In general, increases in ATTD range from 1.04% to 12.09% between the different amino acids, while for AID, increases range from 2.38% to 10.02%. The greatest increases in the ATTD were observed for glycine (12.09%), tyrosine (10.62%), and cysteine (10.23%), while for AID the greatest increases occurred with alanine (10.02%) and cysteine (9.91%).

**Table 3.** Coefficients of ATTD) and AID of amino acids of SBM with or without dietary protease

Amino acids	ATTD^†^			AID^†^		
Protease, ppm	0	200	SEM	0	200	SEM^*a*^
Alanine	0.710	0.751	0.0093	0.767^b^	0.844^a^	0.0092
Arginine	0.796^b^	0.866^a^	0.0084	0.869^b^	0.933^a^	0.0135
Aspartic acid	0.879	0.901	0.0085	0.880	0.884	0.0173
Cysteine	0.592^b^	0.652^a^	0.0121	0.816^b^	0.896^a^	0.0199
Glutamic acid	0.705	0.719	0.0098	0.814	0.831	0.0138
Glycine	0.806^b^	0.903^a^	0.0091	0.868^b^	0.928^a^	0.0139
Histidine	0.779^b^	0.828^a^	0.0091	0.850	0.862	0.0108
Isoleucine	0.836^b^	0.873^a^	0.0039	0.856 ^b^	0.894^a^	0.0119
Leucine	0.827	0.847	0.0060	0.825	0.833	0.0166
Lysine	0.889^b^	0.926^a^	0.0124	0.862^b^	0.930^a^	0.0129
Methionine	0.785^b^	0.834^a^	0.0057	0.851^b^	0.893^a^	0.0132
Phenylalanine	0.865	0.879	0.0039	0.832^b^	0.880^a^	0.0156
Proline	0.775^b^	0.820^a^	0.0082	0.759^b^	0.817^a^	0.0114
Serine	0.854	0.884	0.0065	0.797	0.822	0.0259
Threonine	0.857^b^	0.876^a^	0.0076	0.788^b^	0.852^a^	0.0103
Tyrosine	0.784^b^	0.868^a^	0.0098	0.824	0.843	0.0137
Valine	0.836^b^	0.870^a^	0.0070	0.817^b^	0.880^a^	0.0111
Total AA	0.793^b^	0.833^a^	0.0081	0.818^b^	0.868^a^	0.0145
Total × ileal^‡^	0.813^B^			0.848^A^		

^†^Different lower case letters within a row indicate significantly different means by least significant difference test (*P* < 0.05).

^‡^Different capital letters in the line indicate significantly different means by *F* test (*P* < 0.05). The data is from 10 replicates.

^*a*^Standard error of the means.

For FFSM, the dietary protease increased (*P* < 0.05) the ATTD and AID of all amino acids, except tyrosine (ATTD and AID), arginine, aspartic acid, isoleucine, tyrosine, and valine (only AID) (Table 4). Increases in ATTD range from 3.13% to 11.32% between the different amino acids, while for AID, increases range from 1.73% to 7.29%. The amino acids that had greater increases with the use of the enzyme for the ATTD were the glutamic acid (11.3%), alanine (9.92%), proline (9.33%), and valine (8.05%), and for AID the cysteine (7.29%), proline (5.12%), glutamic acid (4.6%), and glycine (4.35%).

**Table 4.** Coefficients of ATTD and AID of amino acids of FFSM with or without dietary protease

Amino acids	ATTD^†^			AID^†^		
Protease, ppm	0	200	SEM	0	200	SEM^*a*^
Alanine	0.734^b^	0.812^a^	0.0059	0.779^b^	0.796^a^	0.0060
Arginine	0.817^b^	0.874^a^	0.0028	0.888	0.891	0.0028
Aspartic acid	0.849^b^	0.909^a^	0.0122	0.910	0.928	0.0120
Cysteine	0.796^b^	0.837^a^	0.0122	0.792^b^	0.849^a^	0.0124
Glycine	0.897^b^	0.942^a^	0.0055	0.889^b^	0.928^a^	0.0117
Glutamic acid	0.617^b^	0.686^a^	0.0039	0.722^b^	0.755^a^	0.0038
Histidine	0.812^b^	0.854^a^	0.0029	0.899^b^	0.923^a^	0.0028
Isoleucine	0.826^b^	0.881^a^	0.0058	0.863	0.864	0.0060
Leucine	0.836^b^	0.884^a^	0.0068	0.894^b^	0.922^a^	0.0071
Lysine	0.899^b^	0.927^a^	0.0071	0.902^b^	0.935^a^	0.0072
Methionine	0.809^b^	0.862^a^	0.0082	0.850^b^	0.874^a^	0.0080
Phenylalanine	0.874^b^	0.908^a^	0.0066	0.934^b^	0.951^a^	0.0067
Proline	0,790^b^	0.864^a^	0.0061	0.838^b^	0.881^a^	0.0059
Serine	0.846^b^	0.889^a^	0.0037	0.900^b^	0.932^a^	0.0035
Threonine	0.805^b^	0.867^a^	0.0082	0.881^b^	0.919^a^	0.0080
Tyrosine	0.886	0.899	0.0051	0.946	0.957	0.0049
Valine	0.798^b^	0.862^a^	0.0071	0.758	0.762	0.0069
Total AA	0.817^b^	0.868^a^	0.0064	0.862^b^	0.886^a^	0.0068
Total × ileal	0.843^B^		0.0065	0.874^A^		0.0048

^†^Different lower case letters within a row indicate significantly different means by least significant difference test (*P* < 0.05).

^‡^Different capital letters in the line indicate significantly different means by *F* test (*P* < 0.05) The data is from 10 replicates.

^*a*^Standard error of the means.

## DISCUSSION

The dietary protease supplementation was efficient in to increase the use of amino acids for broilers. To the corn, in average, an increase of 5.59% and 4.63% for ATTD and AID, respectively. For SBM, these increases were 6.58% and 6.82% and for FFSM the increase was 6.49% and 3.67%, respectively. The chemical composition of the feedstuffs used in the present study are similar to those shown in the feed composition tables (NRC, 1994; Rostagno et al., 2017), except to the CP content of the FFSM, that was lower than that observed in the literature. Besides good source of AA, the FFSM has a high fat content which may influence the effects of exogenous protease (Duke and Evanson, 1972; Li and Sauer, 1994).

The protease used in the present study is a serine hydrolase. The increase of digestibility of this amino acid was observed only in the corn and in the FFSM. This result may be related to the lower content of this amino acid contained in these feeds (2.9% in the corn, 14.0% in the FFSM compared with 24.1% in the SBM). In these cases, the effects of dietary protease were more effective. Greater protease effect was also observed by Dessimoni et al. (2019) when it reduced dietary protein levels.

The addition of the enzyme increased the total digestibility of both fecal and ileal amino acids in all the feedstuffs studied. Other studies have also indicated the effectiveness of this enzyme in increasing the total digestibility of the amino acids of corn (Bertechini et al., 2009) and of diets based on corn-soybean meal (Angel et al., 2011). In the previous paper, the authors reduced the dietary CP levels by 10% in relation to the control diet and observed the same performance of the birds when compared to the control group.

In general, the effects of dietary protease were different on each amino acid and on each feedstuff. The most significant effects of the enzyme were observed in FFSM and followed by SBM and corn. At FFSM, 70% of amino acids had increase in the ileal digestibility values, 65% of amino acids in the SBM and only 41% of amino acids contained in the corn. Only cysteine, glycine, proline, and threonine had increases in digestibility in all feedstuffs, regardless of the method of determination of digestibility (ATTD or AID). Although glycine, threonine, and proline are present in large amounts in the intestinal fluids, there was an increase in the fecal and ileal digestibility of these nutrients. Glycine corresponds to about 90% of the total amino acids secreted in the bile (Souffrant, 1991), whereas 95% of the intestinal mucus is composed by glycoprotein rich in threonine, serine and proline (Neutra and Forstner, 1987). This result shows that the relationship between digestible amino acids can be altered with the use of exogenous enzymes in the diet.

In this sense, several studies have shown that dietary amino acids can modify the metabolic changes in the organisms. Grimble et al. (1992) indicated that cysteine, a sulfur amino acid, is of prime importance in facilitating increases in liver glutathione (GSH), Zn and protein content in rats. In broilers, Tsiagbe et al. (1987) indicated that cysteine is an important nutrient for the immune response, modulating lymphocyte, and macrophage functions (Droge et al., 1991). Furthermore, feeding l-cysteine increases tissue GSH level, which may be beneficial for growth of chicks reared in conditions of oxidative stress (Tsiagbe et al., 1987). By other hand, excess levels of cysteine and homocysteine can induce tibial dyschondroplasia in broiler chicks. Thus, studies with exogenous enzyme supplementation in broilers are need.

**Figure 1.**
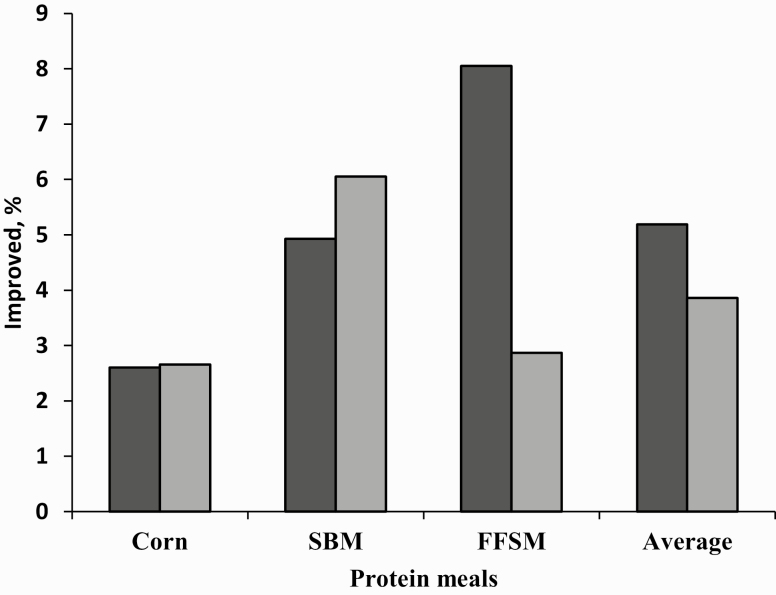
Protease effect (%) on ATTD and AID of the feed ingredients.

When the ATTD technique was applied, the major effects of the protease were for FFSM, followed by SBM and corn (Figure 1). With respect to the AID technique, it was verified that the FFSM presented a smaller increment in relation to the SBM. This result may be related to the higher fiber content contained in the SBM (6.5% vs. 3.4% contained in the FFSM) or the oil contained in the FFSM (18.4% vs. 3.8% for corn and 1.2% for SBM).

Higher values of ileal digestibility of amino acids were observed with FFSM (87.4% vs. 85.2% for corn and 84.3% for SBM). This result may be related to the higher oil content contained in FFSM. Hulan and Bird (1972) reported that the increase of the dietary fat influences the activities of protease, lipase, and amylase in pancreatic juice. Duke and Evanson (1972) reported that fats are powerful inhibitors of gastric emptying. According to Li and Sauer (1994), the delay of gastric emptying also delay the passage of the digestion through the small intestine, increasing the digestibility of the nutrients. However, in the present study there were no differences between fecal and ileal digestibilities when considering total amino acids. Similar results were found by Honda et al. (2009; 2010) working with roosters fed on diets containing fats ranging from 3% to 10%.

The use of nutritional matrices with average increments for all amino acids is an inconsistency, since enzymes are specific and increase the digestibility of amino acids differently. The ideal is to use a nutritional matrix that contemplates the differences in the action of the enzyme in each amino acid.

The ileal digestibility over total is adopted to minimize the effects of the microbiota present in the large intestine (Ten Doeschate et al., 1993). However, the use of the ileal digestibility technique is costly and, in most cases, involves the sacrifice of large numbers of birds. Thus, studies of ileal and fecal digestibility of each amino acid in birds are important so that future studies can establish the relationships between the different values and be able to predict the values of ileal digestibility from fecal digestibility.

## CONCLUSION

The use of dietary protease influences in a different way the total and ileal digestibility of amino acid in feedstuffs for broilers. Most evident effects are observed with higher crude protein feedstuffs. The digestibility of cysteine, glycine, proline, and threonine are more influenced by the addition of this exogenous protease in the diet. The mean of protease effects for all amino acids and in all ingredients studied were 5.19% and 3.86% for ATTD and AID, respectively. The total collection method was lower by 3.7% of amino acids digestibility than the ileal method for vegetal ingredients.
